# Limited accuracy of dose calculation for large fields at deep depths using the BrainSCAN v5.21 treatment‐planning system

**DOI:** 10.1120/jacmp.v6i2.1999

**Published:** 2005-05-21

**Authors:** Wen C. Hsi, Yunkai Zhang, Michael C. Kirk, Damian Bernard, James C.H. Chu

**Affiliations:** ^1^ Departments of Medical Physics and Radiation Oncology Rush University Medical Center 1653 West Congress Parkway Chicago Illinois 60612 U.S.A.

**Keywords:** radiation treatment‐planning system, BrainSCAN, BrainLAB, quality assurance

## Abstract

The Varian 120 multileaf collimator (MLC) has a leaf thickness of 5 mm projected at the isocenter plane and can deliver a radiation beam of large field size (up to 30 cm) to be used in intensity‐modulated radiotherapy (IMRT). Often the dose must be delivered to depths greater than 20 cm. Therefore, during the commissioning of the BrainSCAN v5.21 or any radiation treatment‐planning (RTP) systems, extensive testing of dose and monitor unit calculations must encompass the field sizes (1 cm to 30 cm) and the prescription depths (1 cm to 20 cm). Accordingly, the central‐axis percent depth doses (PDDs) and off‐axis percentage profiles must be measured at several depths for various field sizes. The data for this study were acquired with a 6‐MV X‐ray beam from a Varian 2100EX LINAC with a water phantom at a source‐to‐surface distance (SSD) of 100 cm. These measurements were also used to generate a photon beam module, based on a photon pencil beam dose‐calculation algorithm with a fast‐Fourier transform method. To commission the photon beam module used in our BrainSCAN RTP system, we performed a quantitative comparison of measured and calculated central‐axis depth doses and off‐axis profiles. Utilizing the principles of dose difference and distance‐to‐agreement introduced by Van Dyk et al. [Commissioning and quality assurance of treatment planning computers. Int J Radiat Oncol Biol Phys. 1993; 26:261—273], agreements between calculated and measured doses are <2% and <2 mm for the regions of low‐ and high‐dose gradients, respectively. However, large errors (up to ~5% and ~7% for 20‐cm and 30‐cm fields, respectively, at the depth 20 cm) were observed for monitor unit calculations. For a given field size, the disagreement increased with the depth. Similarly, for a given depth the disagreement also increased with the field size. These large systematic errors were caused by using the tissue maximum ratio (TMR) in BrainSCAN v5.21 without considering increased field size as depth increased. These errors have been reported to BrainLAB.

PACS number: 87.53.‐j

## I. INTRODUCTION

The commercial BrainSCAN v5.21 (BrainLAB AG, Munich, Germany) radiation treatment‐planning (RTP) system has been routinely used in association with the BrainLAB micro multileaf collimator (MLC) (3 mm minimum leaf width and 10 cm maximum leaf position projected at isocenter) for treatment planning with narrow radiation beams (typically ü9 cm field size and ü20 cm patient thickness) for intensity‐modulated radiosurgery (IMRS).^(1)^ With a Varian 120 MLC (5 mm minimum leaf width and 14.5 cm maximum leaf position project at isocenter) in the 6‐MV X‐ray beam of a Varian 2100EX LINAC (Varian Medical Systems, Palo‐Alto, CA), radiation beams of large field sizes (up to $30\times 40{\;\text{cm}}^{2}$) can be used in intensity‐modulated radiotherapy (IMRT) or 3D radiation therapy for delivering dose to prescription points at depths greater than 20 cm. Therefore, during commissioning of the BrainSCAN v5.21 RTP system for treatment planning with a Varian 120 MLC, extensive dosimetry data for various field sizes (1 cm to 30 cm) and prescription depths (1 cm to 20 cm) in a water phantom were acquired to ensure the accuracy of the dose and monitor unit (MU) calculations for the BrainSCAN RTP system.

The central‐axis percent depth doses (PDD) and off‐axis profiles were measured with a water phantom at a source‐to‐surface distance (SSD) of 100 cm. Quantitative comparisons between the measured and calculated distributions were performed using the concept of dose difference and distance‐to‐agreement criteria (<2% and <2 mm for the regions of low‐ and high‐dose gradients, respectively), introduced by Van Dyk et al.^(2)^ The MUs calculated from the BrainSCAN RTP system for delivering 100 cGy to various prescription depths at 100 cm source‐to‐axis distance (SAD) with different field sizes were compared with extracted values obtained from measured output factors. Measured output factor is defined as the ratio of the chamber reading of a given field size and depth to the reading of a $10\times 10{\;\text{cm}}^{2}$ field and a depth at $d_{\text{max}}$ with the same delivered MUs at 100 cm SAD setup for both measurements. Our LINAC was calibrated to deliver 100 cGy per 100 MUs to the depth of $d_{\text{max}}$ in water of a $10\times 10{\;\text{cm}}^{2}$ field. Therefore, to deliver 100 cGy to a prescription point with a defined field size and depth, the required MU value is equal to 100 divided by the measured output factor for the defined field size and depth.

The results of quantitative comparisons of percent depth doses, off‐axis profiles, and MU calculations are presented. Systematic errors in the dose and MU calculations were analyzed to identify the implementation error in the BrainSCAN dose calculation algorithm.

## II. MATERIALS AND METHODS

### A. Beam data measurements

With the Advanced Radiation Measurements, Inc., water phantom scanner, the central‐axis PDDs of 1 cm, 2 cm, 4 cm, 6 cm, 8 cm, 10 cm, 14 cm, 20 cm, and 30 cm square fields at 100 cm SSD were measured at depths up to 28 cm with a water tank phantom $\left(60\times 60\times 50{\;\text{cm}}^{3}\right)$ in a 6‐MV photon beam. The field size was defined with the same opening for the MLCs and secondary collimator jaws. The field size for the MLC or the jaw was calibrated against the light field. A PTW Freiburg sealed chamber and an Exradin A16 micro chamber were used for the measurements. To reduce the influence of detector size^(3)^ for small fields, the PDDs for fields ü10 cm were measured with the micro chamber. To avoid stem/cable effects for large fields, the PDDs for fields >10 cm were measured with the sealed chamber.

With the same phantom setup, the transverse off‐axis percentage profiles for 6‐cm and 20‐cm square fields at depths of 1.4 cm, 5 cm, 10 cm, and 20 cm in directions along and perpendicular to the leaf movement of the MLC were measured. Each field size in the profile measurements was defined by the MLC, while the jaws were set at 24 cm. Similar to the PDD measurements, the smaller and larger fields were measured with micro and sealed chambers, respectively. To obtain the radial factor function used to correct the off‐axis dependence of photon fluence, diagonal cross‐field profiles of a 40‐cm square field, defined by the jaws with retracted MLC, were measured with the sealed chamber at depths of 0.5 cm, 1.4 cm, 2.5 cm, 5 cm, 10 cm, 20 cm, and 30 cm.

The output calibration of the LINAC, that is, the delivered dose (in units of cGy) at the depth of $d_{\text{max}}$ in water per MU by a radiation of $10\times 10{\;\text{cm}}^{2}$ field size, was performed according to the AAPM TG‐51 protocol. Total scatter factors, including the components of the collimator and phantom, at a depth of 5 cm with the water phantom at 100 cm SSD, were measured for various square fields defined by the MLC or jaws. Measurements for square fields ü6 cm were measured with the micro chamber, while measurements for fields >6 cm were measured with the sealed chamber. To evaluate the MU calculation from the BrainSCAN RTP system, the output factors were measured with a water tank $\left(60\times 60\times 50{\;\text{cm}}^{3}\right)$ and a solid water phantom (Gammex RMI 457, 30‐cm thick of $30\times 30{\;\text{cm}}^{2}$ slabs) for square fields of 1 cm, 2 cm, 4 cm, and 6 cm at depths of 1.4 cm, 5 cm, 10 cm, and 20 cm. Although the physical density of this solid water phantom is 1.04 g/cm^3^, its electron density is only 1.011±0.005 g/cm^3^ with respect to water. Since the 6‐MV beams are mainly attenuated through Compton scattering, which is proportional to the electron density of the used material, the effective depth correction for the solid water phantom with respect to the water phantom should be small.

### B. Dose‐calculation algorithm

The photon dose‐calculation algorithm used in the BrainSCAN v5.21 RTP system is a pencil beam dose computation algorithm, which was introduced^(4)^ and developed^(5)^ by Mohan et al. and implemented by BrainLAB. For a collimator with an arbitrary shape, the ideal dose distribution, *IDD*(*x,y,d*), at depth *d* in an infinite water phantom is calculated by a 2D convolution of the polyenergetic pencil beam kernel, $D_{p}(x-x\prime ,y-y\prime ,d)$, and the photon fluence, xxphi(x′,y′,d):

(1) $$IDD(x,y,d)=\iint \phi (x^{\prime },y^{\prime },d)\times D_{p}(x-x^{\prime },y-y^{\prime },d)dx^{\prime }dy^{\prime }.$$

Since $D_{p}(x-x\prime ,y-y\prime ,d)$ is based on a generic spectrum, an optional source function correction is applied to take into account the machine‐specific adaptations (such as the influence of the finite size of the source, collimator, and flattening filter scatter, curvature of the leaf ends, and other smearing effects). A radial factor function is applied on the photon fluence to account for the off‐axis dependence. The 2D integration is then calculated using Fourier transform algorithms.

Dose to a point inside a patient by a shaped beam is calculated from

(2) $$\begin{array}{rl} & dose(x,y,d)=\text{MU}\times M_{Nlin}\times S_{t}(c_{MLC},c_{jaw})\times {TMR}_{PB}(\min(c_{MLC},c_{jaw}),r_{rad})\times \\ & {\left(\frac{SID}{SSD+d}\right)}^{2}\times IDD(x^{\prime },y^{\prime },r_{rad}) \end{array}$$

where $r_{\text{rad}}$ is the radiological path length of the beam from the tissue surface to the calculation point, corrected for tissue density inhomogeneities. BrainSCAN precalculates the *IDD*(*x,y,d*) of each individual field at the source isocenter distance (SID) in water. The IDD is dependent on the $r_{\text{rad}}$ and lateral distances at $x\prime =x\left(\frac{\text{SID}}{\text{SSD}+d}\right);y\prime$ is defined similarly. The depth dependence of the calculated dose is then recalibrated with respect to the measured beam data by $S_{t}{\;\text{x TMR}}_{\text{PB}},$, where the total scatter factor $S_{t}$ describes the relative output factor, and the tissue maximum ratio, ${\text{TMR}}_{\text{PB}}$, is calculated from the measured percentage depth doses at 100 cm SSD. Since mainly narrow fields are considered in the BrainSCAN RTP software for IMRS treatment, ${\text{TMR}}_{\text{PB}}$ is independent of SSD and can be obtained by the following transformation equation:

(3) $${TMR}_{PB}(c,d)={\left(\frac{SSD+d}{SSD+d_{cal}}\right)}^{2}\times PDD(c,d),$$

where *c* is the collimator opening, *d* is the depth, and $d_{\text{cal}}$ is the calibration depth of the nominal output factor $\left(M_{Nlin}\right)$, which is used for obtaining the absolute delivered dose. An adaptive grid algorithm is used to accelerate the dose calculations with a coarse grid in the regions of smooth dose distributions and a fine grid in the regions where the dose distributions are inhomogeneous, such as in the penumbra region.

## III. RESULTS

In comparing off‐axis percentage profiles, the calculated field widths (at 50% of the central axis value) for the 6‐cm and 14‐cm fields at various depths agreed within 1 mm with the measured widths. The percentage differences between the measured and calculated values are <2% and <4% of the $d_{\text{max}}$ value for the in‐field and the out‐of‐field regions, respectively. The differences between the calculated and measured penumbra width (defined at 20% to 80%) are <2 mm. The calculated penumbra is steeper and more symmetric at the 50% values than that measured. The BrainSCAN RTP system used a steeper penumbra to compensate for the limited spatial resolution of the scanning ionization chamber on the scanned profiles. This may not alter the dose uniformity inside the planning target volume (PTV) and the dose gradient at the edge of the PTV in an IMRT treatment with multiple X‐ray beams where the dose uniformity and gradient are largely modified by the overlapping of beam. However, the subject of this quantitative difference is not in the scope of this paper.

**Figure 1:**
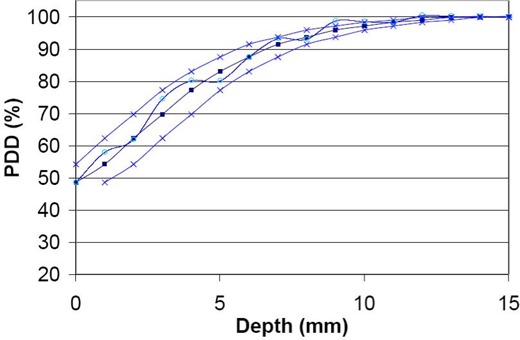
The calculated and measured PDDs for a 10‐cm field size are plotted as diamonds and circles, respectively. Measured PDDs with depths shifted ±1 mm are plotted as crosses.

The method used to evaluate the differences between the calculated and measured central‐axis PDDs with a water phantom at 100 cm SSD is based on (1) the distance to the nearest point where the calculated and measured doses are identical and (2) the percentage difference between the calculated and measured doses at the same measurement point. The standard deviation (1xxsigma)_ percentage and position uncertainties of each measured PDD are ±0.5% and ±0.5 mm, respectively. The position uncertainty of the calculations is ±1.25 mm, while a 2.5‐mm pixel size is used in the pencil beam kernel. The measured and calculated central‐axis PDDs agreed within 1 mm in the buildup region (0.5 cm to 1.5 cm depth) for all fields. A comparison between the measured and calculated values for the 10‐cm field in the buildup region is shown in Fig. 1. The periodic fluctuation observed for the calculated depth dose may be the result of the position uncertainty in the pencil beam kernel. For depth doses between 1.5 cm and 25 cm, the difference between the calculated and measured values is less than 2% of the $d_{\text{max}}$ value for all field sizes.

After validating the depth doses and the off‐axis profiles with the phantoms scanned at 100 cm SSD, the accuracy of the MU calculations for a point at 100 cm SAD was examined for various depths and field sizes. The comparisons of the calculations with the measurements performed with water and solid water phantoms are shown in Table 1 for various depths and field sizes. Although the effective depth correction for the solid water phantom with respect to the water phantom should be small (about 1%) as observed for depths less than 10 cm, differences of about 2% were observed at a depth of 20 cm for various field sizes. Therefore, the effective depth correction should be taken into account when using a solid water phantom in measurements at large depths.

**Table 1:** Extracted monitor units (MUs) from measured output factors with water and solid water phantoms, and MUs calculated by the BrainSCAN RTP for delivering 100 cGy to prescription depths of various field sizes

field defined by jaws	15×15	15×15	15×15	15×15	15×15	30×30	30×30
field defined by MLC	1×1	2×2	4×4	6×6	10×10	20×20	30×30
Dmax calculated	125	110	105	101	99	95	94
extracted (water)	124.3	110.6	104.6	101.6	98.8	93.5	92.8
(ext.–cal)/cal*100(%)	−0.56	0.53	−0.35	0.55	−0.21	−1.59	−1.30
extracted (solid water)	124.5	110.6	104.5	101.5	99.0	93.8	93.2
(ext.–cal)/cal*100(%)	−0.41	0.57	−0.45	0.52	−0.03	−1.29	−1.81
5 cm calculated	145	126	116	111	107	100	99
extracted (water)	144.5	126.6	116.8	112.0	106.9	100.2	98.6
(ext.–cal)/cal*100(%)	−0.32	0.46	0.71	0.88	−0.03	0.20	−0.46
extracted (solid water)	144.9	126.2	116.8	111.2	107.1	100.1	99.0
(ext.–cal)/cal*100(%)	−0.05	0.18	0.67	0.22	0.07	0.05	0.0
10 cm calculated	183	157	142	133	125	112	108
extracted (water)	183.3	159.4	144.5	136.3	127.5	114.9	111.7
(ext.–cal)/cal*100(%)	0.18	1.50	1.79	2.48	2.00	2.59	3.43
extracted (solid water)	185.3	160.1	145.6	136.2	127.5	115.4	112.3
(ext.–cal)/cal*100 (%)	1.24	2.00	2.51	2.38	2.00	3.07	4.00
20 cm calculated	287	247	221	203	183	154	141
extracted (water)	293.2	252.6	227.7	210.5	189.7	161.4	151.5
(Ext.–Cal)/Cal*100(%)	2.15	2.28	3.01	3.69	3.70	4.81	7.42
extracted (solid water)	298.5	255.8	229.7	213.2	193.0	164.5	155.2
(Ext.–Cal)/Cal*100(%)	4.02	3.57	3.96	5.02	5.44	6.85	10.04

Since the water phantom was used to calculate MUs in BrainSCAN, MUs extracted from measured output factors with the water phantom were used to evaluate the MU calculation of the BrainSCAN RTP system. The difference between the calculated and measured MUs increases with depth for a given field size and with field size for a given depth. Large errors of ~5% and ~7% were observed for 20‐cm and 30‐cm fields, respectively, at a 20‐cm depth.

## IV. DISCUSSION

Although the measured and calculated PPDs and the percentage profiles with a 100‐cm SSD geometry agree to less than 2% or within 1 mm, we observed large differences between the calculated and extracted MUs from measurements with the water phantom. Examination of the dose calculation algorithm used in BrainSCAN reveals an error in converting the measured PPDs to TMRs in the BrainSCAN RTP system. We found that the change of field size was not taken into account correctly in Eq. (3). Percent depth doses from larger field sizes were used to calculate the TMRs. As a result, the calculated values of TMR in BrainSCAN are overestimated.

**Figure 2:**
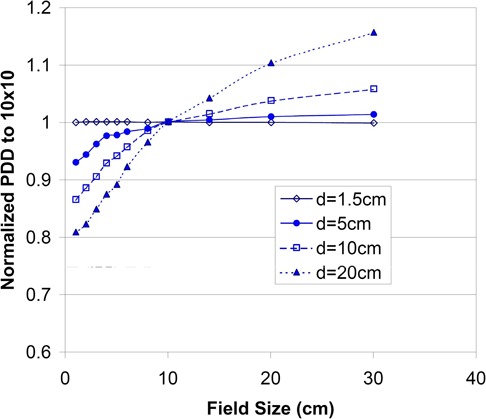
The measured PDDs as a function of field size for various depths at 100 cm SSD. PDDs are normalized with respect to the measured PDD of a $10\times 10{\;\text{cm}}^{2}$ field for each depth.

The correct transformation function^(6)^ for Eq. (3) should be

(4) $${TMR}_{PB}(r_{d},d)={\left(\frac{SSD+d}{SSD+d_{cal}}\right)}^{2}\times PDD(r,d,SSD)\times \frac{S_{p}(r_{t0})}{S_{p}(r_{d})},$$

where $r_{t0}(\text{calculated as}\;(r{\;x(\text{SSD}+t}_{0})/\text{SSD}))$ is the field size at reference depth $t_{0}$. The $S_{p}$ is the phantom scatter factor. The field size $r_{d}(\text{calculated as}\;c\;x\;(\text{SSD}+d)/100)$ increases linearly with the depth *d* and increases with field size *c* of the collimator (MLC/jaws) opening. The inverse square correction is taken into account by the term ${\left(\frac{\text{SSD}+d}{\text{SSD}+d_{\text{cal}}}\right)}^{2}$. To estimate the errors in calculating the TMR value using Eq. (3), measured depth doses were normalized with respect to a 10‐cm field size at each depth for the various depths shown in Fig. 2. The change in field size from the surface to a 20‐cm depth for a small 4‐cm field is less than 1 cm, but for a large 20‐cm field, defined at the surface, the field size is 24 cm at a 20‐cm depth. The values of PDDs at a 20‐cm depth change about 5% between the field sizes of 20 cm and 24 cm, as shown in Fig. 2. Therefore, using a PDD of field size of 24 cm instead of 20 cm in Eq. (3) to calculate the TMR, as is calculated in BrainSCAN, overestimates the TMR by about 5%. Since the PDDs increase for larger field sizes, and the slope of increments is steeper for larger depths as shown in Fig. 2, the errors resulting from using Eq. (3) instead of Eq. (4) increase for deeper depths and larger field sizes. In addition, the phantom scatter factor $S_{p}$ also introduces errors (typically about 2% to 3%).

With the correct ${\text{TMR}}_{\text{PB}}$, the dose^(6)^ is calculated by

(5) $$\begin{array}{rl} & dose(x,y,d)=MU\times M_{Nlin}\times S_{c}(c_{MLC},c_{jaw})\times S_{p}(r_{d})\times {TMR}_{PB}(r_{d},r_{rad})\times \\ & {\left(\frac{SID}{SSD+d}\right)}^{2}\times IDD(x^{\prime },y^{\prime },r_{rad}), \end{array}$$

where the collimator scatter factor $S_{c}$ depends only on the collimator opening. The correct formulas for both TMR and dose calculations have been reported to BrainLAB.

## Supporting information

Supplementary Material Files

Supplementary Material Files

## References

[1] Monk JE , Perks JR , Doughty D , Plowman PN . Comparison of a microleaf collimator with a 5‐mm‐leaf‐width collimator for intracranial stereotactic radiotherapy. Int J Radiat Oncol Biol Phys. 2003;57:1443–1449. 14630284 10.1016/s0360-3016(03)01579-7

[2] Van Dyk J , Barnett RB , Cygler JE , Shragge PC . Commissioning and quality assurance of treatment planning computers. Int J Radiat Oncol Biol Phys. 1993;26;261–273. 8491684 10.1016/0360-3016(93)90206-b

[3] Sibata CH , Mota HC , Beddar AS , Higgins PD , Shin KH . Influence of detector size in photon beam profile measurements. Phys Med Biol. 1991;36; 621–631. 2068227 10.1088/0031-9155/36/5/005

[4] Mohan R , Chui C , Lidofsky L . Differential pencil beam dose computation model for photon. Med Phys. 1985;13;64–73. 10.1118/1.595924 3951411

[5] Mohan R , Chui C‐S . Use of fast Fourier transforms in calculating dose distributions for irregularly shaped field for the three‐dimensional treatment planning. Med Phys. 1986;14;70–77. 10.1118/1.596097 3104741

[6] Khan FM . The physics of radiation therapy. 3rd ed. Philadelphia (PA): Lippincott Williams & Wilkins; 2003 p 178.

