# Supplementary figures and images for: Limited accuracy of dose calculation for large fields at deep depths using the BrainSCAN v5.21 treatment‐planning system
Source: J Appl Clin Med Phys. 2005 May 21;6(2):12–8. doi: 10.1120/jacmp.v6i2.1999 (PMC5723477; doi:10.1120/jacmp.v6i2.1999)

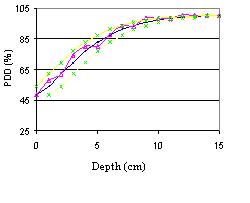

Supplement: Supplementary file 1 — Supplementary Material Files [file ACM2-6-12-s001.jpg]

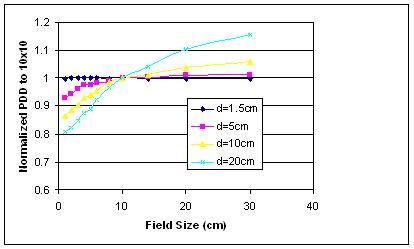

Supplement: Supplementary file 2 — Supplementary Material Files [file ACM2-6-12-s002.jpg]
